# Supplementary material for: A protocol for an assessment of the health information system in Khomas region, Namibia
Source: PLoS One. 2025 Sep 23;20(9):e0306939. doi: 10.1371/journal.pone.0306939 (PMC12456804; doi:10.1371/journal.pone.0306939)
Supplement: Attachment — Submitted filename: Review of Manuscript Number PONE-D-24-25668.pdf [file pone.0306939.s001.pdf]

<u>Review of Manuscript Number PONE-D-24-25668</u>

Dear Authors,

Thank you for the opportunity to review this manuscript titled "An assessment of the Health Information System in Khomas region, Namibia". In this manuscript, the authors introduce their study proposal towards research that aims to assess human factors affecting the Health Information System (HIS) in Namibia. Given challenges of a low resource setting, they aim to evaluate progress of the HIS from 2012-2022, against the 2012 USAID recommendations. The topic is essential and of particular interest to both practitioners and policy-makers today as it concerns the exponential implementations of HIS in the health sector. Although intended to better patient management, these implementations come with their own set of challenges.

Overall, the paper makes for a clear and eloquent read, with plans of the study protocol clearly documented. The authors have provided adequate supporting background literature from countries with similar contexts in the region. There is clarity in the chosen framework and findings from previous studies, forming a baseline for comparison in the future. The authors mention three distinct objectives and a detailed methods section on how they plan to achieve these objectives.

The paper, however, requires much work to be considered further for publishing. Some of my suggestions are in keeping with PlosOne's study protocol guidelines https://journals.plos.org/plosone/s/submission-guidelines#loc-study-protocols. Although this protocol is not for a clinical trial or systematic review, I recommend that the authors refer to the guidelines to enhance the quality of their submission. My feedback and queries for clarification are as follows:

1. **Title**: It is recommended that the title of the manuscript have the word 'protocol' in it (please see guidelines).

2. **Background:**
    a. The protocol claims gaps in literature pertaining to human factors that influence HIS efficiency. It would help the reader to know what authors mean by *human factors*? Will they be, for example, adopting findings from the cited Nikol et al. 2013 paper that mentions human factors? If not defined before, what is the authors' working operational definition of human factors? Previous studies, like Kremer et al. 2019 and Hudson, D. 2023, may be considered as example of human factors, for instance.
    b. Although aims and objectives have been mentioned, what is the study's main research question?

3. **Materials and Methods:**
    a. *Conceptual framework:* Referring to the use of the PRISM framework, it will help to understand whether the study will be a mere replication of the studies done in other countries because there is no prior work done in Namibia? What about the context in Namibia is 'intriguing' or demands such a study be replicated? How are findings from this study expected to add value to the contributions already made?

b. *Multi-method approach*: Please provide a citation for the approach used.

c. *The sample size calculation:*
   - The number of participants calculated for the questionnaire has been demonstrated well. "306 participants will be randomly recruited". Please elaborate on this. Consider specifying clearly that the sample size calculation was only for the larger number of nurses. The questionnaire would be offered to all of the remaining HIS staff. And why this is the case.
   - How was the number of participants for key informant interviews derived or pre-determined to be 17?

d. *Data saturation:* please mention how data saturation will be determined for the qualitative part of the study.

e. *Informed consent:* The protocol has mentioned that informed consent will be obtained. How will this be done? (For example, through a patient information sheet, language etc.)

f. *Data collection tools:* Will the interviews to address Objective 3 be structured, semi-structured, unstructured? What are some topics that will be covered in these? Will the authors be submitting a copy of the checklists, interview guides etc. used for data collection?

g. *Data analysis:* Outline some potential assumptions and explicitly describe what aspects of the proposed analyses are exploratory. The protocol mentions that a "document review checklist has been developed, which includes four categories". It will be valuable for the reader to know what these (working) category names are.

4. **Pilot study:** How will the authors consider modifications, if required, from the feedback during the pilot study?

5. **Ethical considerations:** Please include a note on consent taking for all participates. How will consent be taken? What contingency steps will be considered if there are refusals?

6. **Discussions:** Consider enhancing the discussions by addressing: what contributions do the authors expect from their study? Which ongoing discussions and debates around HIS will benefit from your empirical findings? Will there be any contributions towards the conceptual framework?

7. **Proof reading suggestions:**

a. Citations: In Introduction, revise line 8 citations to read as either "1,3" or "1-3".

b. Some sentences are densely structured and may require to be revised with adequate connecting words. Wherever possible, these lines are highlighted. Please consider revising these lines for simplicity:
   - Introduction – last line; "Despite the assistance… staff to retain them".
   - Background – last line in 1st paragraph; "Other challenges highlighted… computers and internet".

c. Objective 2: "A quantitative of… (MOHSS). Please revise grammar of sentence.

d. Objective 2: “The sample size calculation depicted in Figure 1”. Please change this to Figure 2 for Sample size calculation. Also, please provide reference for the formula used for the estimation.
e. Acronyms: Please check that acronyms are used after full form presented. For example, MOHSS has been repeated in full form several times.

Once again, thank you for the opportunity to review this paper. I wish the authors all the very best in their research.

References:

- Hudson D. Physician engagement strategies in health information system implementations. Healthc Manage Forum. 2023 Mar;36(2):86-89. doi: 10.1177/08404704221131921. Epub 2022 Oct 31. PMID: 36314071; PMCID: PMC9975817.
- Kremer L, Leeser L, Breil B. Mental Workload Relating Health Information System - A Literature Review. Stud Health Technol Inform. 2019 Sep 3;267:289-296. doi: 10.3233/SHTI190840. PMID: 31483284.
- Nicol E, Bradshaw D, Phillips T, Dudley L. Human Factors Affecting the Quality of Routinely Collected Data in South Africa [Internet]. Ebooks.iospress.nl. 2013. Available from: https://ebooks.iospress.nl/publication/34107
