# Supplementary material for: A protocol for an assessment of the health information system in Khomas region, Namibia
Source: PLoS One. 2025 Sep 23;20(9):e0306939. doi: 10.1371/journal.pone.0306939 (PMC12456804; doi:10.1371/journal.pone.0306939)
Supplement: Attachment — Submitted filename: Review of resubmit R2.pdf [file pone.0306939.s004.pdf]

Review of Manuscript Number PONE-D-24-25668_R2

Dear Editor,

Thank you for this third opportunity to review this manuscript now titled "An assessment of the Health Information System in Khomas region, Namibia: Study Protocol".

A very minor modification to expand an acronym in the manuscript was required from my end. The authors have fulfilled this.

However, a matter of concern may be the authors' statement that the comments from Reviewer 4 do not match with their study topic.

Not much more from my side. I wish the authors all the very best.

Many thanks again for the opportunity.
