# Supplementary material for: A protocol for an assessment of the health information system in Khomas region, Namibia
Source: PLoS One. 2025 Sep 23;20(9):e0306939. doi: 10.1371/journal.pone.0306939 (PMC12456804; doi:10.1371/journal.pone.0306939)
Supplement: Attachment — Submitted filename: Review of Manuscript ID PONE-D-24-25668 Responses.docx [file pone.0306939.s003.docx]

5 December 2024

Plos One journal

Dear Reviewers

**AN ASSESSMENT OF THE HEALTH INFORMATION SYSTEMS IN KHOMAS REGION, NAMIBIA: Study protocol**

I wish to submit my moderated protocol for the above study.

All requested comments have been addressed on pages two to eight of this document by including a table with the comments and the responding changes made, as well as the page numbers on which the changes can be found.

Yours sincerely

Prof Edward Nicol

| **COMMENTS** | **RESPONSES** | **PAGE** |
| --- | --- | --- |
|  |  |  |
| 1. Title: It is recommended that the title of the manuscript have the word ‘protocol’ in it (please see guidelines). | The word protocol has been added to the title. The title is “An assessment of the Health Information System in Khomas region, Namibia: Study protocol” | 1 |
| 2. Background:  a. The protocol claims gaps in literature pertaining to human factors that influence HIS efficiency. It would help the reader to know what authors mean by human factors? Will they be, for example, adopting findings from the cited Nikol et al. 2013 paper that mentions human factors? If not defined before, what is the authors’ working operational definition of human factors? Previous studies, like Kremer et al. 2019 and Hudson, D. 2023, may be considered as example of human factors, for instance.  b. Although aims and objectives have been mentioned, what is the study’s main research question? | Background  a. The human factors will be partly adopted from the findings cited in Nicol et al, 2013 paper. The other human factors are also adopted from some papers in Namibia; Blodlo & Hamunyela 2017, Kapepo & Yashik, 2018 and Nengomasha et al, 2018.  These human factors are defined and described in the background, paragraph one, second and third sentences, as well as paragraphs 3 and 4.  b. Thank you for this comment. The research questions are listed below and have been included in the text.  **Research Questions**  1. Has there been any improvement in the health information system in Khomas region in terms of human resource capacity (skills and competencies) since 2012?  2. What are the behavioral and organizational factors influencing the performance of health information systems in Khomas region?  3. What are the barriers and facilitators affecting the implementation of recommendations aimed to improve the NHIS? | 4,5-6  6 |
| 3. Materials and Methods:  a. Conceptual framework: Referring to the use of the PRISM framework, it will help to understand whether the study will be a mere replication of the studies done in other countries because there is no prior work done in Namibia? What about the context in Namibia is ‘intriguing’ or demands such a study be replicated? How are findings from this study expected to add value to the contributions already made?  b. Multi-method approach: Please provide a citation for the approach used.  c. The sample size calculation:   1. The number of participants calculated for the questionnaire has been demonstrated well. “306 participants will be randomly recruited”. Please elaborate on this. Consider specifying clearly that the sample size calculation was only for the larger number of nurses. The questionnaire would be offered to all of the remaining HIS staff. And why this is the case. 2. How was the number of participants for key informant interviews derived or pre-determined to be 17?   d. Data saturation: please mention how data saturation will be determined for the qualitative part of the study.  e. Informed consent: The protocol has mentioned that informed consent will be obtained. How will this be done? (For example, through a patient information sheet, language etc.)  f. Data collection tools: Will the interviews to address Objective 3 be structured, semi-structured, unstructured? What are some topics that will be covered in these? Will the authors be submitting a copy of the checklists, interview guides etc. used for data collection?  g. Data analysis: Outline some potential assumptions and explicitly describe what aspects of the proposed analyses are exploratory. The protocol mentions that a “document review checklist has been developed, which includes four categories”. It will be valuable for the reader to know what these (working) category names are. | The extent to which the 2012 recommendations from USAID have been implemented and their impact on RHIS improvement remain unclear. Additionally, most evaluations of the Namibian HIS have not thoroughly investigated the human factors influencing it ^2,10-11,13,15^. The proposed study aims to address gaps in HIS by utilizing the Performance of Routine Information System Management (PRISM)’s Organizational and Behavioural Assessment Tools (OBAT) alongside qualitative methods.  How it will add value to the contributions is already addressed under Discussions, data dissemination, paragraph two.  b. Citation for the Multi method approach added.  Thank you for this comment. The following paragraph has been added to provide more clarity on the selection process:  “Using the staff registers obtained from the district office, the 306 health workers will be proportionally drawn from each of the 14 health facilities included in the study. Within each facility, a systematic sampling technique with a random starting point will be applied to select the required number of healthcare workers. Additionally, all 24 data management staff will be included in the study. If a selected staff member is unavailable, the research team will proceed to the next available individual on the list, continuing this process until the target number is achieved.  Our choice of 17 participants was based on the following literature that show that code and meaning saturations can be reached with between 16 and 24 interviews. Hennink MM, Kaiser BN, Marconi VC. Code saturation versus meaning saturation: how many interviews are enough? Qual Health Res. 2017;27(4):591–608.  Guest B, Bunce A, Johnson L. How many interviews are enough? An experiment with data saturation and variablility. Field Methods. 2006;18(1):59–82.  Thanks. A paragraph on data saturation has been included in the text.  e. Highlighted under Ethical considerations: Informed consent, both verbal and written, will be obtained from participants of the OBAT survey and in-depth interviews. For the survey, consent will be sought electronically through an introductory page outlining the study details, followed by a consent statement that participants must agree to by clicking a checkbox or button before proceeding. Consent for the in-depth interviews will be obtained verbally, the consent process will be recorded, including explicit permission for audio recording.  f. Addressed under objective 3: The Semi structured in-depth interviews will be conducted face-to-face or virtually with 17 key informants from health facilities and the Health Information and Research Directorate using an interview schedule.  The document review checklist and interview guide, as well as the links to the OBAT survey and modified facility checklist will be shared by the authors on request.  g. Thank you for the comment. This paragraph has been added to the text.  “These categories are as follows: availability of an HR information system (electronic or manual), HIS policies and strategic plans, HIS committee and directorate, and the availability of support services such as study leave, loans, and bursaries.” | 6  8 and 19  12  12  15  16  14  11 |
| 4. Pilot study: How will the authors consider modifications, if required, from the feedback during the pilot study? | The purpose of the pilot is to test the suitability of the questionnaires and identify whether further adjustments are needed. The pilot phase will also help in gauging the time required to complete the questionnaire.  After the pilot, revisions will be made to the questionnaire where necessary, and lessons learnt will be adapted accordingly.  See text on page 16 regarding the amendment. | 16 |
| 5. Ethical considerations: Please include a note on consent taking for all participates. How will consent be taken? What contingency steps will be considered if there are refusals? | Thanks. This has already been addressed under the ethical considerations paragraph: | 16 |
| 6. Discussions: Consider enhancing the discussions by addressing: what contributions do the authors expect from their study? Which ongoing discussions and debates around HIS will benefit from your empirical findings? Will there be any contributions towards the conceptual framework? | Thank you for your comment. This manuscript is a protocol for a proposed study. The discussions will be more detailed after data collection analysis have been completed and results available. |  |
| 7. Proof reading suggestions:  a. Citations: In Introduction, revise line 8 citations to read as either “1,3” or “1-3”.  b. Some sentences are densely structured and may require to be revised with adequate connecting words. Wherever possible, these lines are highlighted. Please consider revising these lines for simplicity:   Introduction – last line; “Despite the assistance… staff to retain them”.   Background – last line in 1st paragraph; “Other challenges highlighted… computers and internet”.  c. Objective 2: “A quantitative of… (MOHSS). Please revise grammar of sentence.  d. Objective 2: “The sample size calculation depicted in Figure 1”. Please change this to Figure 2 for Sample size calculation. Also, please provide reference for the formula used for the estimation.  e. Acronyms: Please check that acronyms are used after full form presented. For example, MOHSS has been repeated in full form several times. | Thank you  a. rectified to 1-3  b. the introduction has been revised.  “Despite support from these agencies, low- and middle-income countries (LMICs) still face challenges in implementing key recommendations. These recommendations focus on clearly defining staff roles and structures, particularly for Health Information Systems (HIS), providing ongoing training for staff, and offering incentives to retain personnel.”.  Background- sentence revised  “Other issues include unintegrated data systems, lack of system monitoring and evaluation, limited resources to support and sustain implemented systems, political barriers, absence of policies; and inadequate infrastructures such as computers and internet”  c. Thank you. The sentence has been reworded accordingly as follows:  “Data will be collected quantitatively using a self-administered questionnaire adapted from the PRISM’s Organizational and Behavioural Assessment Tool (OBAT). The questionnaire will be administered to participants from health facilities in the Khomas region and the Health Information and Research Directorate (HIRD) at the MoHSS. It will assess participants’ behaviours, attitudes, and knowledge, as well as identify factors influencing the performance of the NHIS in the Khomas region.”  d. Addressed. Figure 1 changed to figure 2. Two references for the sample size have been inserted.  e. Acronyms have been addressed as suggested | Page 4  Page 4  5  11, 12  12 and 21 |
