# Supplementary material for: A protocol for an assessment of the health information system in Khomas region, Namibia
Source: PLoS One. 2025 Sep 23;20(9):e0306939. doi: 10.1371/journal.pone.0306939 (PMC12456804; doi:10.1371/journal.pone.0306939)
Supplement: Attachment — Submitted filename: Review of Manuscript ID PONE-D-24-25668 Responses26feb25.docx [file pone.0306939.s005.docx]

26 February 2025

Plos One journal

Dear Editor,

**A PROTOCOL FOR AN ASSESSMENT OF THE HEALTH INFORMATION SYSTEMS IN KHOMAS REGION, NAMIBIA:**

We wish to submit my moderated protocol for the above study.

We appreciate that Reviewer #3 is satisfied with our responses to the initial comments and have further addressed the minor comment raised.

However, it appears there may have been a mix-up with the comments from Reviewer #4. These comments and feedback seem to pertain to a different manuscript—specifically, a study conducted in Ethiopia—rather than our submission, "An Assessment of the Health Information System in Khomas Region, Namibia: Study Protocol." The comments do not appear to be relevant to our study.

Could you kindly review this and provide clarification?

Thank you for your time and assistance.

Yours sincerely

Prof Edward Nicol

| **COMMENTS** | **RESPONSES** | **PAGE** |
| --- | --- | --- |
|  |  |  |
| **Reviewer #3:**   1. Dear Authors,  Thank you for the second opportunity to review this manuscript now titled “An assessment of the Health Information System in Khomas region, Namibia: Study Protocol”.  I am satisfied with the responses to my review comments and amendments made to the paper before resubmission.  A very minor modification to the manuscript required is to expand the acronym HR in the Introduction.  I refer back to the editor for the final decision regarding this paper and wish the authors all the very best in the next stage of their research. | Thank you. The acronym HR has been written in full as suggested | 9 |
| **Reviewer #4:**  Overall, this manuscript presents valuable insights into healthcare professionals' intentions regarding digital health data hubs in Ethiopia. The topic of digital health data hubs is highly relevant, especially in the context of achieving Sustainable Development Goals (SDGs). Your focus on Ethiopia adds an important dimension to the global conversation about digital health. These merits contribute to making your manuscript a valuable addition to the literature on digital health adoption among healthcare professionals, particularly in developing countries like Ethiopia.  General Feedback 1. Clarity and Conciseness: a. The abstract is informative but could be more concise. Aim to summarize key findings in fewer words while maintaining clarity  b. Some sentences are complex, consider breaking them into shorter sentences for better readability.  Example from the introduction Original: "Gradually, more and more people are using mobile devices and the Internet and intend to use them for healthcare services, as digital health (DH) has significantly accelerated the achievement of the Sustainable Development Goals (SDGs) and strengthened healthcare systems in Africa."  Suggested Revision: "More people are gradually using mobile devices and the Internet for healthcare services. Digital health (DH) has significantly accelerated progress toward achieving the Sustainable Development Goals (SDGs) and strengthening healthcare systems in Africa."  Example from Background Section: Original: "To date, it is of great importance to examine the emerging cloud-based adoption at the organizational level Healthcare organizations have continually recorded data over time for customers, suppliers, and stakeholders to analyze the data and derive insights."  Suggested Revision: "It is important to examine emerging cloud-based adoption at the organizational level. Healthcare organizations have continually recorded data over time for customers, suppliers, and stakeholders to analyze this information and derive insights."  2. Structure: a. Ensure that each section flows logically into the next. For example, the transition from the introduction to methods could be smoother by summarizing how the background leads to your research questions or hypotheses.  b. Consider using subheadings within sections (e.g., "Methods," "Results") to improve navigation through the document.  3. Methodology: a. It might be helpful to provide more detail about how you ensured the validity and reliability of your survey instruments beyond mentioning pre-testing and PCA.  b. Clarify how you addressed potential biases in sampling or data collection.  4. Results Presentation: a. Present results in a clear manner using tables or figures where appropriate (e.g., showing demographic data).  5. Discussion: a. The discussion should connect back to your research questions/hypotheses more explicitly.  b. Consider discussing limitations earlier in this section rather than at the end; this can help contextualize your findings.  6. Conclusion: Your conclusion summarizes findings well but could benefit from a stronger emphasis on implications for practice and future research directions.   Specific Feedback Abstract: a. The phrase "the health system has a unified digital health center" might need clarification - does it mean there is one central hub for all data?  b. Instead of stating "this study aims," use past tense since this is a completed study. "This study assessed..."  Introduction a. The introduction provides good context but could be streamlined by focusing on key points relevant to your study's objectives.  b. Avoid excessive citations in introductory paragraphs; instead, synthesize information from multiple sources into cohesive statements.  Methods a. Specify what type of healthcare professionals were surveyed (e.g., doctors, nurses) early on for context.  b. In describing SEM analysis, briefly explain why this method was chosen over others.  Results a. Include descriptive statistics before diving into inferential statistics to give readers context about your sample.  b. Be cautious with terms like “significant” without specifying p-values initially; clarify what constitutes significance based on your analysis plan.  Discussion a. Expand on how these findings relate to existing literature—what do they add or challenge?  b. Discuss practical applications of your findings more thoroughly—how can stakeholders implement changes based on this research?   Congratulations to the team on this great achievement. | **Not applicable to how study** | 6 |
