# Supplementary material for: A protocol for an assessment of the health information system in Khomas region, Namibia
Source: PLoS One. 2025 Sep 23;20(9):e0306939. doi: 10.1371/journal.pone.0306939 (PMC12456804; doi:10.1371/journal.pone.0306939)
Supplement: Attachment — Submitted filename: Response to Reviewers.docx [file pone.0306939.s006.docx]

**COVER LETTER**

**RESPONSE TO REVIEWERS**

***Manuscript ID: PONE-D-24-25668R2*,**

**A protocol for an assessment of the Health Information System in Khomas region, Namibia.**

| **Reviewer comments** | **Page #** |
| --- | --- |
| Dear Academic Editor and reviewers,  The authors would like to thank the reviewers for reading through this manuscript. We sincerely appreciate the feedback we received on this manuscript. We have addressed the main concern raised by the reviewers, as is reflected below, and revised the manuscript accordingly.  Thank you for the opportunity to revise this submission.  Regards,  Edward Nicol |  |
| **EDITOR**  Along with your revision, please be sure to include a cover letter that outlines point-by-point how you address all of the reviewer’s comments. Your revision should also carefully follow the journal style as presented in the Instructions for Authors available at the journal website.  A few things that I tell all authors at this stage:  Be sure to double space the paper throughout, including references, but not include extra spaces. |  |
| **Reviewer #3**  Thank you for this third opportunity to review this manuscript now titled “An assessment of the Health Information System in Khomas region, Namibia: Study Protocol”. I have no new comments to make. A very minor modification to expand an acronym in the manuscript was required from my end. The authors have fulfilled this.  However, a matter of concern may be the authors’ statement that the comments from Reviewer 4 do not match with their study topic.  Not much more from my side. I wish the authors all the very best. Many thanks again for the opportunity.  Thank you for the compliment and the constructive comments. |  |
| **Reviewer #4**  Thank you for submitting this well‑conceived study protocol assessing human and organizational determinants of HIS performance in Namibia’s Khomas region. The strengths include the use of the internationally recognized PRISM framework, a multi‑method approach combining qualitative and quantitative data, and clear alignment of objectives with data collection tools.  Thank you.  Major points to address prior to publication:   1. Explicit Research Questions & Hypotheses: Translate each objective into one or more focused research questions or hypotheses. State whether analyses are confirmatory or exploratory, and align hypotheses accordingly.   Thank you for your comment, however, the research questions were already included in the revised submission (see page 6). The research questions are both confirmatory and exploratory, aiming to identify if there has been an improvement since 2012 in terms of the human capacity in the Khomas region. And also, to explore behavioural and organisational factors influencing the performance of the HIS system. These are also highlighted on pages 10, 11, and 14.   2. Statistical Power & Analytical Plan: Provide a formal power calculation (e.g., minimum detectable effect size at α = 0.05, 80% power), or justify the adequacy of n = 330 for planned analyses.   The following paragraphs have been included to justify the sample size calculation:  “Sample size estimation was conducted with statistical consultation. The target population comprised 1,513 healthcare workers in the Khomas region, including 1,489 nurses (522 enrolled nurses and 967 registered nurses) and 24 data management staff (15 clerks, 5 HIS officers, and 4 departmental staff). The minimum required sample was calculated using the formula for an infinite population where ‘n’ is the required sample size, ‘Z’ is the z-score at the desired confidence level (1.96 for 95%), 𝑝 is the estimated proportion (0.5 for maximum variability), and 𝑒 is the margin of error (0.05). Substituting these values yields a required sample of 384 (Figure 1). The finite population correction was then applied where N=1,513. This adjustment produced a final sample size of approximately 306 nurses. To ensure representativeness, all 24 data management staff were included, resulting in a total of 330 participants.”  Describe how potential confounders will be controlled for in the ANOVA or through additional covariate analyses.  “This will enable the assessment of effects of professional cadre and facility type on OBAT scores while adjusting for potential confounders such as years of experience in the health system, level of formal training in HIS, type of facility (clinic vs. hospital) and Highest education attained.”  3.      Data Sharing Details: Specify the target public repository (with DOI if known) and the types of data files to be deposited (e.g., .csv, .sav for survey data; transcripts or codebooks for qualitative data).  The reprint of this study will be made available at: doi: https://doi.org/10.1101/2024.06.28.24309648  Unfortunately, the datasets generated and analysed during this study will not be publicly shared in a repository in order to protect participants’ privacy and maintain the confidentiality of the Namibian health information system. However, data access will be considered upon a request to the authors. The requests will be reviewed in accordance the ethics of the Namibian Ministry of Health and Social Services.  4.      Manuscript Editing: Engage a professional copy‑editor to correct grammatical errors and improve flow. Ensure all figures and tables are properly embedded with visible captions.  Thank you for this suggestion. The manuscript has been edited accordingly  Minor suggestions: - Clarify the timeline of data collection and analysis in a brief Gantt chart or timeline paragraph.  Thank you. A Gantt chart has been included as an appendix as suggested  - Define any acronyms upon first use (e.g., “M&E”).  Addressed  - In the Ethics section, specify how storage and destruction of audio recordings comply with local data protection laws beyond POPIA.  c) All audio recordings are stored on a password protected laptop; and they will be permanently deleted upon completion of the research. These are all in line with the ethical guidelines of the Namibian Ministry of Health and Social Services, as well as the Stellenbosch university ethical guideline, in addition to the POPIA.  - Repetition & Formatting: The Data Availability question is duplicated in the submission form.  Addressed  - Grammar & Style: Instances of missing articles (“A functional HIS involve…” should be “A functional HIS involves…”), subject–verb disagreement (“These aids come in the forms…” → “These aids come in the form…”), and inconsistent tense.  Addressed  - Typos & References: Reference numbering sometimes jumps (e.g., “1.3”), and spacing around citations is inconsistent. Figure captions refer to “Figure 1” but the embedded image link is not visible in the manuscript PDF.  Addressed  - Clarity: Long paragraphs (especially in the Introduction) should be broken up and streamlined  References for “other models” have been included as suggested  Recommendation: A careful copy‑edit is needed to correct grammatical errors, ensure consistent referencing, and improve overall readability before peer review.  Done | All  all  all |
